# VaMIEL1-mediated ubiquitination of VaMYB4a orchestrates cold tolerance through integrated transcriptional and oxidative stress pathways in grapevine

**DOI:** 10.1093/hr/uhaf093

**Published:** 2025-03-22

**Authors:** Yaping Xie, Kai Lv, Qinhan Yu, Jieping Wu, Junxia Zhang, Huixian Zhao, Junduo Li, Ningbo Zhang, Weirong Xu

**Affiliations:** College of Enology and Horticulture, Ningxia University, No.498 Helanshan West Street, Xixia District, Yinchuan, Ningxia 750021, China; College of Enology and Horticulture, Ningxia University, No.498 Helanshan West Street, Xixia District, Yinchuan, Ningxia 750021, China; School of Life Sciences, Ningxia University, No.498 Helanshan West Street, Xixia District, Yinchuan, Ningxia 750021, China; College of Enology and Horticulture, Ningxia University, No.498 Helanshan West Street, Xixia District, Yinchuan, Ningxia 750021, China; College of Enology and Horticulture, Ningxia University, No.498 Helanshan West Street, Xixia District, Yinchuan, Ningxia 750021, China; College of Enology and Horticulture, Ningxia University, No.498 Helanshan West Street, Xixia District, Yinchuan, Ningxia 750021, China; College of Enology and Horticulture, Ningxia University, No.498 Helanshan West Street, Xixia District, Yinchuan, Ningxia 750021, China; College of Enology and Horticulture, Ningxia University, No.498 Helanshan West Street, Xixia District, Yinchuan, Ningxia 750021, China; Engineering Research Center of Grape and Wine, Ministry of Education, Ningxia University, No.498 Helanshan West Street, Xixia District, Yinchuan, Ningxia 750021, China; Key Laboratory of Modern Molecular Breeding for Dominant and Special Crops in Ningxia, No.498 Helanshan West Street, Xixia District, Yinchuan 750021, China; State Key Laboratory of Efficient Production of Forest Resources, No.498 Helanshan West Street, Xixia District, Yinchuan 750021, China; College of Enology and Horticulture, Ningxia University, No.498 Helanshan West Street, Xixia District, Yinchuan, Ningxia 750021, China; School of Life Sciences, Ningxia University, No.498 Helanshan West Street, Xixia District, Yinchuan, Ningxia 750021, China; Engineering Research Center of Grape and Wine, Ministry of Education, Ningxia University, No.498 Helanshan West Street, Xixia District, Yinchuan, Ningxia 750021, China; Key Laboratory of Modern Molecular Breeding for Dominant and Special Crops in Ningxia, No.498 Helanshan West Street, Xixia District, Yinchuan 750021, China; State Key Laboratory of Efficient Production of Forest Resources, No.498 Helanshan West Street, Xixia District, Yinchuan 750021, China

## Abstract

Cold stress poses a significant threat to viticulture, particularly under the increasing pressures of climate change. In this study, we identified *VaMIEL1*, a RING-type E3 ubiquitin ligase from *Vitis amurensis*, as a negative regulator of cold tolerance. Under normal temperature conditions, VaMIEL1 facilitates the ubiquitination and subsequent proteasomal degradation of the cold-responsive transcription factor VaMYB4a, thereby attenuating its regulatory role in the *CBF-COR* signaling cascade. However, under cold stress, *VaMIEL1* expression is downregulated, leading to the stabilization of VaMYB4a and the activation of *CBF-COR* signaling. Through a combination of biochemical assays and functional analysis in *Arabidopsis thaliana* and grapevine calli, we demonstrate that *VaMIEL1* overexpression reduces cold tolerance, as evidenced by increased oxidative stress, excessive reactive oxygen species (ROS) accumulation, and downregulated expression of cold-responsive genes. Conversely, silencing of *VaMIEL1* enhances cold tolerance by stabilizing VaMYB4a and boosting antioxidant defenses. These findings uncover a previously unrecognized regulatory mechanism by which VaMIEL1 modulates cold tolerance through transcriptional and oxidative stress pathways, offering potential targets for the development of climate-resilient grapevine cultivars and other crops.

## Introduction

Plants, as sessile organisms, are constantly exposed to various environmental challenges, with extreme temperatures representing one of the most significant stressors [1, 2]. Cold stress, in particular, imposes severe limitations on plant growth, development, and productivity, especially for crops such as grapevine (*Vitis vinifera* L.) [3]. Cold stress negatively impacts the geographic distribution, yield, and fruit quality of temperate crops [4]. With the ongoing challenges posed by climate change, developing cold-tolerant crops is critical for both agriculture and food security [5, 6]. Understanding the molecular mechanisms that underlie plant cold tolerance is thus of paramount importance.

At the cellular level, plants employ sophisticated mechanisms to perceive and adapt to cold stress [7, 8]. Central to these responses is the ubiquitin–proteasome system (UPS), a highly conserved pathway responsible for selective protein degradation [9, 10]. This system allows plants to rapidly eliminate misfolded or damaged proteins, maintain cellular homeostasis, and modulate regulatory proteins in response to environmental changes [10–12]. E3 ubiquitin ligases, as key components of the UPS, confer substrate specificity by targeting proteins for ubiquitination and subsequent degradation. These ligases regulate critical stress-response pathways by controlling transcription factors, enzymes, and signaling proteins [13, 14].

Recent studies have highlighted the pivotal role of E3 ubiquitin ligases in cold tolerance. In *Arabidopsis thaliana*, the RING-type E3 ligase HOS1 targets the transcription factor ICE1 for degradation, thus negatively regulating the *CBF-COR* cold-response pathway and reducing freezing tolerance [15, 16]. In apple (*Malus domestica*), the U-box E3 ligase *MdPUB23* similarly mediates cold sensitivity by promoting the degradation of MdICE1 [17, 18]. However, despite these advances in model systems and some fruit crops, the molecular mechanisms by which E3 ligases regulate cold tolerance in grapevine remain largely unexplored.

While considerable progress has been made in understanding how E3 ligases modulate cold stress in model plants, several questions remain unresolved, particularly in perennial species like grapevine. First, it is unclear whether similar regulatory mechanisms operate in *Vitis amurensis*, a wild grape species naturally adapted to cold climates. While some studies suggest a functional conservation of E3 ligases across plant species, the specific roles of these ligases in perennial crops are poorly defined [19, 20]. Additionally, the extent to which E3 ligases regulate MYB transcription factors in cold stress responses remains ambiguous. While MYB factors are well known to modulate abiotic stress responses, their regulation via ubiquitination has been underexplored in grapevine. Another area of debate concerns the crosstalk between post-translational modifications, such as ubiquitination, and transcriptional regulation during cold stress adaptation. In many plants, the precise timing and regulation of protein degradation are critical for an effective stress response. However, the interaction between these pathways in grapevine, particularly in relation to cold stress, remains poorly characterized [2, 21].

**Figure 1:**
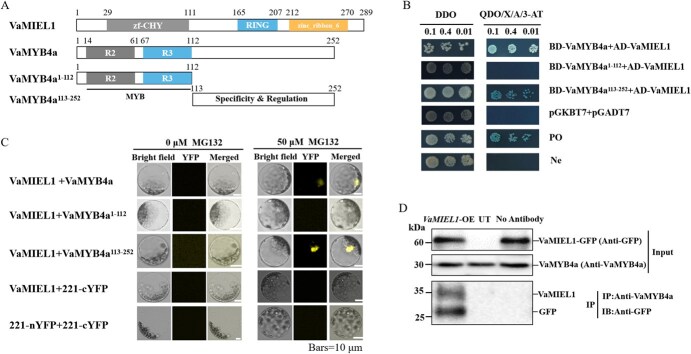
Interaction between VaMIEL1 and VaMYB4a. (A) Schematic representation of VaMIEL1 and VaMYB4a protein structures, including deletion constructs. VaMIEL1 contains a zinc finger (zf-CHY) and RING finger domain, while VaMYB4a features R2 and R3 MYB domains. (B) Y2H assay was employed to assess the interactions between VaMIEL1 and VaMYB4a. Yeast strain growth was observed on double dropout (DDO:SD/−Trp/−Leu) and quadruple dropout (QDO/X/A/3-AT:SD/−Leu/−Trp/-His/−Ade/X-α-gal/AbA/3-AT) selective media. For controls, the interaction between mouse p53 and SV40 large T-antigen was used as a positive control (PO), and the lamin C and SV40 large T-antigen interaction as a negative control (Ne). (C) BiFC assay confirmed the *in vivo* interaction between VaMIEL1 and VaMYB4a. VaMIEL1 was fused to the N-terminal YFP fragment (nYFP), while VaMYB4a was linked to the C-terminal YFP fragment (cYFP). The constructs were coexpressed with and without the proteasome inhibitor MG132. Fluorescence, indicating YFP reconstitution, verified the interaction. Negative controls consisted of constructs with unfused nYFP and cYFP fragments. Scale bars = 10 μm. (D) Co-IP assays showing that VaMIEL1-GFP coprecipitates with VaMYB4a. Immunoprecipitations (IP) were performed with anti-VaMYB4a antibody, and immunoblotting (IB) was conducted using anti-GFP antibody. UT, untransformed calli. ‘No antibody’ denotes the absence of MYB4a antibody immunoprecipitation.

Our previous work identified *VaMYB4a* as a positive regulator of cold stress and uncovered its candidate interactor, VaMIEL1, through yeast two-hybrid (Y2H) screening [22]. Here, we investigate the role of the E3 ubiquitin ligase VaMIEL1 in modulating cold tolerance by examining its interaction with VaMYB4a and its downstream effects on the *CBF-COR* signaling pathway. Using *A. thaliana* and grapevine (*V. vinifera* L.) calli as models, we assess the impact of *VaMIEL1* overexpression and silencing on cold stress responses, with a focus on the transcriptional regulation of cold-responsive genes. By revealing a regulatory mechanism in which *VaMIEL1*mediates the degradation of VaMYB4a, our study provides new insights into the role of the UPS in cold tolerance regulation in perennial species. These findings could inform breeding strategies to enhance cold tolerance in grapevines, contributing to the development of climate-adaptive crops.

## Results

### VaMIEL1, an E3 ubiquitin ligase, interacts with VaMYB4a both *in vitro* and *in vivo*

Our previous work has demonstrated that *VaMYB4a* enhances cold tolerance in grapevine by upregulating cold-responsive genes within the *CBF-COR* signaling pathway [22]. Building on this foundation, we sought to explore the functional significance of VaMYB4a's interaction with VaMIEL1, an E3 ubiquitin ligase identified through Y2H screening, and to elucidate the mechanistic interplay between these two proteins under cold stress conditions.

To map the specific region of VaMYB4a responsible for its interaction with VaMIEL1, two truncated forms of VaMYB4a were generated: VaMYB4a^1-112^, containing the N-terminal MYB DNA-binding domains (1-112 aa), and VaMYB4a^113-252^, comprising the C-terminal regulatory domain (113-252 aa) (Fig. 1A). Y2H assays were performed using these truncated constructs and the full-length VaMYB4a. Yeast strains cotransformed with BD-VaMYB4a (or its truncated variants) and AD-VaMIEL1 were serially diluted and plated on selective media to assess interaction strength. As shown in Fig. 1B, robust growth was observed across all dilutions for the full-length VaMYB4a and the C-terminal VaMYB4a^113-252^ construct, indicating a strong interaction with VaMIEL1. In contrast, the N-terminal fragment (VaMYB4a^1-112^) failed to support interaction. Control vectors (pGBKT7 + pGADT7, pGBKT7-p53 + pGADT7-T, and pGBKT7-Lam + pGADT7-T) displayed the expected growth patterns, validating assay specificity (Fig. 1B). These results suggest that the C-terminal region of VaMYB4a (113-252 aa) is both necessary and sufficient for mediating the interaction with VaMIEL1.

**Figure 2:**
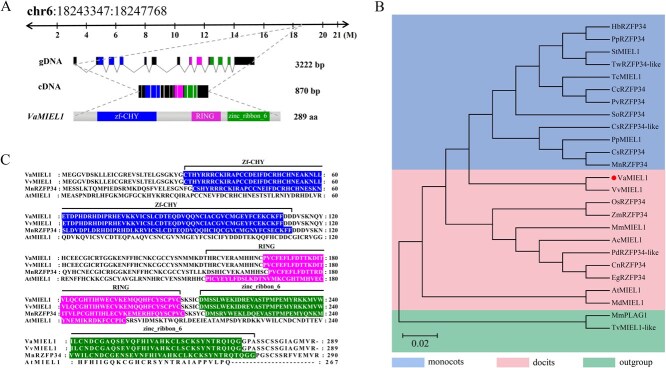
Sequence analysis of *VaMIEL1*. (A) Genomic structure of *VaMIEL1*, displaying the gene’s genomic DNA (gDNA, 3222 bp) and cDNA (870 bp). The corresponding protein structure, consisting of 289 amino acids, includes a zinc finger CHY domain (zf-CHY), a RING domain, and a zinc ribbon domain (zinc_ribbon_6). (B) Phylogenetic analysis of plant MIEL1 proteins. A phylogenetic tree was generated using the maximum likelihood approach with 1000 bootstrap replicates and subsequently visualized in the NCBI Tree Viewer. Tree topology reflects the minimum sum of branch lengths (S), with sequence homology exceeding 75% among the selected sequences. The analysis categorizes sequences into monocots, dicots, and outgroups. (C) Protein sequence alignment of VaMIEL1 (*V. amurensis*: PP471211), VvMIEL1 (*V. vinifera*: XP_002274709.1), MnRZFP34 (*M. notabilis*: XP_024022996.1), and AtMIEL1 (*A. thaliana*: NP_197366.1). The conserved Zf-CHY, RING, and zinc ribbon domains are highlighted to indicate structural similarities across species.

To further verify the interaction between VaMIEL1 and VaMYB4a in planta, bimolecular fluorescence complementation (BiFC) assays were performed. In the absence of the proteasome inhibitor MG132, fluorescence signals were undetectable across both the nucleus and cytoplasm. Upon treatment with 50 μM MG132, however, yellow fluorescence localized to distinct nuclear bodies, indicating that proteasome inhibition alters the localization—and possibly the stability—of the VaMIEL1–VaMYB4a complex (Fig. 1C). Importantly, the truncated VaMYB4a construct lacking the C-terminal region did not exhibit altered localization upon MG132 treatment, reinforcing the critical role of the C-terminal region in mediating both interaction and stability. To confirm the *in vivo* interaction between VaMIEL1 and VaMYB4a, co-immunoprecipitation (Co-IP) assays were performed using transgenic calli overexpressing *VaMIEL1*-GFP (Green Fluorescent Protein). Immunoprecipitation with an anti-VaMYB4a (ABclonal, Wuhan, China, 19000445A-1-2) antibody successfully pulled down GFP-tagged VaMIEL1, confirming the physical interaction between VaMIEL1 and VaMYB4a *in vivo* (Fig. 1D). No signal was detected in control extracts (untransformed or without antibody), demonstrating the specificity of the interaction under the experimental conditions employed.

### Structural and evolutionary characterization of *VaMIEL1* as a conserved E3 ubiquitin ligase in *V. amurensis*

To elucidate the possible role of *VaMIEL1* in enhancing cold tolerance in *V. amurensis*, we performed an in-depth sequence and expression analysis. *VaMIEL1* encodes a protein of 289 amino acids, featuring an open reading frame (ORF) of 870 bp and an estimated molecular weight of 34.68 kDa. The gene is located on chromosome 6, spanning positions 18 243 347–18 247 768, and comprises 13 exons and 12 introns. Structural domain analysis revealed the presence of a ZF-CHY zinc finger domain, a RING finger domain, and a zinc_ribbon_6 domain, all of which are characteristic features of E3 ubiquitin ligases involved in protein ubiquitination and degradation (Fig. 2A).

Phylogenetic analysis based on maximum likelihood methods demonstrated that *VaMIEL1* clusters with dicot homologs, including those from *V. vinifera*, while monocot homologs formed a separate clade. The high bootstrap values supporting these clades highlight the clear evolutionary divergence between monocots and dicots, likely reflecting functional diversification of E3 ubiquitin ligases after speciation (Fig. 2B). To further investigate evolutionary relationships, we performed a comparative sequence analysis of *VaMIEL1* with its homologs from *V. vinifera* (VvMIEL1), *A. thaliana* (AtMIEL1), and *Morus notabilis* (MnRZFP34). This analysis revealed a high degree of conservation within the E3 ligase domains, particularly the RING finger domain, which plays a crucial role in ubiquitin transfer. The conserved cysteine and histidine residues essential for zinc ion coordination were maintained across these homologs, suggesting a conserved mechanism of action (Fig. 2C).

### *VaMIEL1* exhibits tissue-specific expression and dynamic cold-induced regulation through a low-temperature-responsive promoter in *V. amurensis*

**Figure 3:**
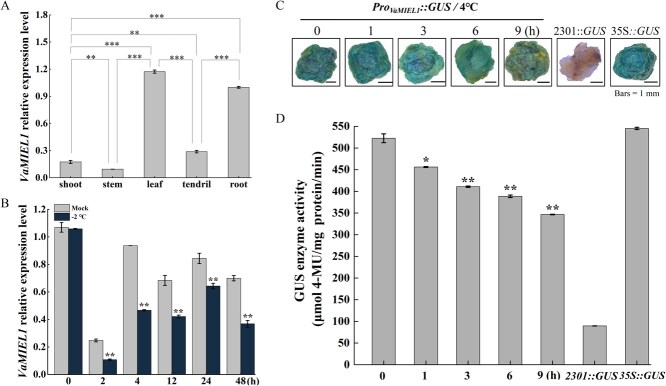
Expression and GUS enzyme activity analysis of *VaMIEL1* in various plant tissues and in response to cold stress. (A) RT–qPCR analysis of *VaMIEL1* expression in various tissues of *V. amurensis* (root, shoot, stem, leaf, and tendril). *VvActin1* was used as an internal reference. Data are expressed as mean ± SD from three biological replicates. Statistical significance was determined by one-way ANOVA, followed by Tukey’s HSD test for comparisons among multiple groups. Pairwise comparisons were performed using an independent *t*-test (^*^P < 0.05, ^**^P < 0.01, ^***^P < 0.001). (B) Temporal expression profile of *VaMIEL1* in the leaves of *V. amurensis* ‘ZuoShan-1’ seedlings subjected to cold stress (−2°C) for varying durations (0–48 h). *VvActin1* served as an internal control. Values represent the mean ± SD of three independent replicates, with significance relative to the 0 h time point assessed by one-way ANOVA and LSD test (^**^P < 0.01). (C) Histochemical GUS staining of calli transformed with the *VaMIEL1* promoter-driven GUS reporter construct (*Pro_VaMIEL1_::GUS*), following exposure to cold stress at 4°C for 0, 1, 3, 6, and 9 h. *CaMV35S::GUS* and *pCAMBIA2301::GUS* served as positive and negative controls, respectively. Scale bar = 1 mm. The images were digitally extracted for visual comparison. (D) GUS activity assay in calli expressing *Pro_VaMIEL1_::GUS* during cold stress exposure. GUS enzyme activity was quantified as 4-MU production (μmol 4-MU/mg protein/min) in *Pro_VaMIEL1_::GUS* transgenic calli at various time points following cold stress (0, 1, 3, 6, and 9 h)*.* Error bars denote the SD of three biological replicates. Statistical significance was determined by *t*-test, comparing each time point (1, 3, 6, 9 h) to the 0-h control (^*^P < 0.05, ^**^P < 0.01).

To assess the tissue-specific expression of *VaMIEL1*, we performed reverse-transcription quantitative polymerase chain reaction (RT-qPCR) across various tissues of *V. amurensis*. Under normal conditions, *VaMIEL1* expression was lowest in stems, while progressively higher expression levels were observed in shoots, tendrils, and leaves, with the highest expression detected in leaves, where it was 1.2-fold higher than in roots (Fig. 3A). In contrast, expression levels in stems, shoots, and tendrils were less than one-third of the root expression level, indicating a distinct tissue-specific expression pattern for *VaMIEL1*.

To further explore the role of *VaMIEL1* in response to cold stress, we monitored its expression over time in the leaves of ‘ZuoShan-1’ seedlings exposed to −2°C for varying durations (Fig. 3B). Notably, *VaMIEL1* expression decreased significantly within the first 2 h of cold treatment, reaching approximately half of the control level. Interestingly, expression levels peaked at 24 h, reaching 0.6-fold of the control level, before declining again. After 48 h of cold exposure, *VaMIEL1* expression was significantly lower than both the pretreatment and mock-treated controls. These findings suggest that *VaMIEL1* is dynamically regulated during cold stress, possibly contributing to the cold acclimation process in *V. amurensis*.

To investigate the molecular response of *VaMIEL1* to cold stress, genomic DNA from *V. amurensis* ‘ZuoShan-1’ was used for PCR amplification, resulting in the isolation of the *ProVaMIEL1* promoter, which is 2451 bp in length. Sequence analysis using the PlantCare database (https://bioinformatics.psb.ugent.be/webtools/plantcare/html/) identified a *Low-Temperature-Responsive* (LTR) element (CCGAAA) within this promoter region (Table S1). The *VaMIEL1* promoter was subsequently cloned into the pCAMBIA2301::GUS vector to generate the *pCAMBIA2301::Pro_VaMIEL1_::GUS* reporter construct, which was transiently expressed in 'Chardonnay' grape calli to evaluate promoter activity under cold stress. Under standard conditions, GUS (*β*-Glucuronidase) activity in *Pro_VaMIEL1_::GUS* calli remained low. However, following exposure to 4°C, a significant increase in GUS staining was observed, indicating that the *VaMIEL1* promoter is activated in response to cold stress (Fig. 3C).

In contrast, the *CaMV35S*::GUS construct, used as a positive control, exhibited high, constitutive expression, while the empty pCAMBIA2301::GUS vector served as a negative control, showing negligible expression. Quantitative analysis of GUS activity further supported these observations, with a marked increase in GUS enzyme activity in *Pro_VaMIEL1_::GUS* calli after 9 h of cold stress (Fig. 3D). Interestingly, prolonged cold exposure of *35S::Pro_VaMIEL1_::GUS* calli at 4°C resulted in a gradual reduction in GUS staining and a significant decrease in GUS activity over time. This suggests that although the *VaMIEL1* promoter is initially responsive to cold stress, its activity may be downregulated as cold treatment continues, implying a dynamic regulatory mechanism controlling *VaMIEL1* expression under prolonged cold stress conditions.

### *VaMIEL1* overexpression impairs cold tolerance in *Arabidopsis* by enhancing oxidative stress and suppressing CBF-COR pathway gene expression

To explore the potential role of *VaMIEL1* in cold tolerance, transgenic *Arabidopsis* lines with overexpression of *VaMIEL1* were generated. Three independent lines were confirmed through PCR, western blot (WB), and RT-qPCR analyses, with the two lines exhibiting the highest expression levels (#2 and #4) selected for cold tolerance assays (Fig. S1A–C). In parallel, we obtained the *AtMIEL1* T-DNA insertional mutant (*miel1*, SALK_087883) from the European *Arabidopsis* Stock Centre (NASC). Genotyping via triprimer PCR confirmed the presence of the T-DNA insertion in the *miel1* mutant (Fig. S1D), and RT-qPCR analysis showed that *AtMIEL1* was expressed in the wild type (WT) but absent in the *miel1* mutant (Fig. S1E).

**Figure 4:**
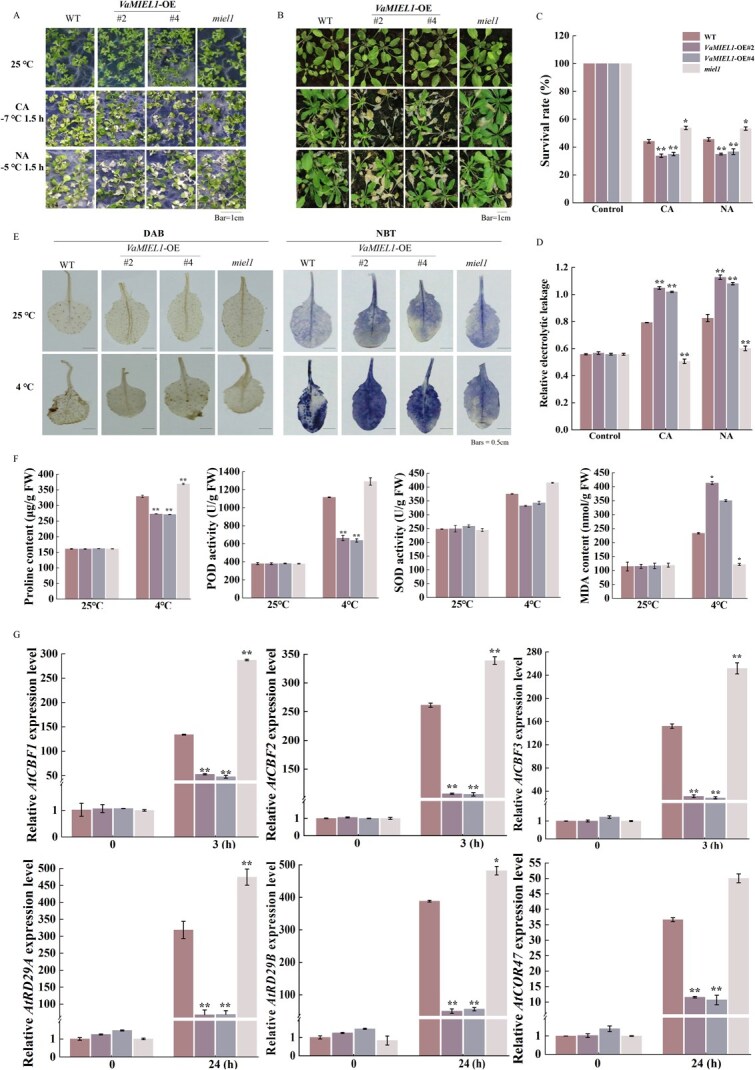
Overexpression of *VaMIEL1* in *Arabidopsis* suppresses cold tolerance. (A) Cold stress phenotypes of *VaMIEL1*-OE (*VaMIEL1* overexpression) *Arabidopsis* seedlings. Twelve-day-old seedlings were grown on one-half MS plates at 22°C before being exposed to CA (−7°C for 1.5 h) or NA (−5°C for 1.5 h) conditions. WT: wild type, #2 and #4: *VaMIEL1*-OE lines, *miel1*: *AtMIEL1* knockout mutant. Scale bars = 0.5 cm. (B) Cold phenotypes of 3-week-old *VaMIEL1*-OE and *miel1* plants grown in soil under similar CA and NA conditions. Scale bars = 0.5 cm. (C) Survival rates of WT, *VaMIEL1*-OE lines (#2 and #4), and *miel1* plants following cold stress. (D) Relative electrolyte leakage in WT, *VaMIEL1*-OE, and *miel1* lines after CA or NA treatments. (E) Histochemical staining with 3,3′-diaminobenzidine (DAB, for H₂O₂ detection) and Nitrotetrazolium blue chloride (NBT, for O_2_^·−^ detection) in leaves of WT, *VaMIEL1*-OE (#2 and #4), and *miel1* plants under control (25°C) or cold stress conditions (4°C for 3 days). Scale bars = 0.5 cm. (F) Quantification of Pro content, MDA content, SOD activity, and POD activity in WT, *VaMIEL1*-OE, and *miel1* mutants following 3 days of cold treatment at 4°C. Data represent the mean ± SD of three independent biological replicates, with 15 plants per replicate. FW, fresh weigh. (G) Expression levels of cold-responsive genes in the *CBF-COR* pathway (*AtCBF1, AtCBF2, AtCBF3, AtCOR47, AtRD29A,* and *AtRD29B*) in WT, *VaMIEL1*-OE (#2 and #4), and *miel1* mutants at 3 and 24 h under control (25°C) or cold stress (4°C) conditions. Gene expression was measured by RT-qPCR, normalized to *AtActin1* as an internal control. Data represent the mean ± SD of three independent experiments. Statistical significance was determined using one-way ANOVA followed by *post hoc* Tukey test (^*^P < 0.05, ^**^P < 0.01).

A comprehensive analysis of cold stress responses was conducted, encompassing phenotypic observations, measurements of cellular damage, reactive oxygen species (ROS) accumulation, biochemical stress markers, and expression profiling of cold-responsive genes. Under non-acclimated (−5°C for 1.5 h) and cold-acclimated (−7°C for 1.5 h) conditions, the *VaMIEL1*-overexpressing (OE) lines (#2 and #4) exhibited heightened sensitivity to cold stress compared to WT. Under one-half Murashige and Skoog (MS) medium, both *VaMIEL1*-OE lines (*VaMIEL1*-OE #2 and #4) exhibited reduced growth and more severe wilting compared to WT plants, suggesting that *VaMIEL1* expression compromises cold tolerance in *Arabidopsis*. In contrast, *miel1* mutants displayed improved performance relative to WT, although no significant growth differences were observed (Fig. 4A). To further investigate cold tolerance under low-temperature stress, 4- to 6-week-old WT, *VaMIEL1*-OE, and *miel1* seedlings were subjected to freezing treatment. The results revealed that *VaMIEL1*-OE plants showed a marked reduction in growth and cold tolerance, while *miel1* mutants exhibited superior growth under cold stress, with fewer desiccated leaves and higher survival rates, indicating enhanced cold tolerance (Fig. 4B). Survival rates of the *VaMIEL1*-OE lines decreased by 53% and 64%, respectively, under these conditions, highlighting the negative impact of *VaMIEL1* overexpression on cold tolerance. Furthermore, electrolyte leakage, an indicator of membrane damage, increased significantly in the *VaMIEL1*-OE lines under cold stress. Electrolyte leakage in lines #2 and #4 increased by 0.3 and 0.2 relative to WT, indicating more severe membrane damage (Fig. 4D). In contrast, the *miel1* mutant displayed reduced electrolyte leakage (0.1 less than WT) and a 15% higher survival rate following cold stress treatment, suggesting enhanced cold tolerance in the absence of *AtMIEL1* (Fig. 4C and D).

Staining with NBT (Nitrotetrazolium Blue chloride) and DAB (3,3′-diaminobenzidine) in *VaMIEL1*-OE lines revealed a marked increase in O_2_^·−^ (superoxide radical) and H_2_O_2_ (hydrogen peroxide) accumulation under cold stress, with staining intensity ~40% higher than in WT plants, indicating an elevated oxidative stress response (Fig. 4E). Biochemical analyses further supported this, as *VaMIEL1*-OE lines exhibited ~50% increase in malondialdehyde (MDA) content, indicative of enhanced lipid peroxidation. In contrast, proline (Pro) levels, a key osmoprotectant, decreased by 20% relative to WT. This was accompanied by a 25% increase in superoxide dismutase (SOD) activity and a 15% reduction in peroxidase (POD) activity, suggesting an imbalance in the antioxidant defense system in the OE lines. Specifically, under cold stress at 4°C, Pro content and POD activity in the OE lines decreased further, alongside reduced SOD activity, providing additional evidence of their compromised stress response. Conversely, the *miel1* mutant exhibited the opposite trend, with elevated Pro levels and increased activities of antioxidant enzymes, alongside a reduction in MDA content (Fig. 4F). These findings suggest that *VaMIEL1* plays a distinct regulatory role in modulating cold tolerance in *Arabidopsis*, contributing to increased oxidative stress and impaired cellular protection mechanisms in the OE lines, while the *miel1* mutant showed enhanced stress resilience.

Gene expression analysis under cold stress conditions revealed significant alterations in the transcriptional levels of key cold-responsive genes within the *CBF-COR* signaling pathway. In *VaMIEL1*-OE lines, expression levels of *AtCBF1, AtCBF2, AtCBF3, AtCOR47, AtRD29A,* and *AtRD29B* were significantly downregulated after 3 and 24 h of exposure to 4°C, with a pronounced suppression of *AtCBF3* at the 3-h time point (Fig. 4G). This downregulation of *CBF* and *COR* genes in the OE lines, compared to WT, highlights the negative regulatory impact of *VaMIEL1* overexpression on cold response signaling. The reduced expression of these key cold-responsive genes underscores the role of *VaMIEL1* as a negative regulator in the cold stress adaptation process in *Arabidopsis*.

### *VaMIEL1* negatively regulates grapevine cold tolerance via the *CBF-COR* signaling pathway

**Figure 5:**
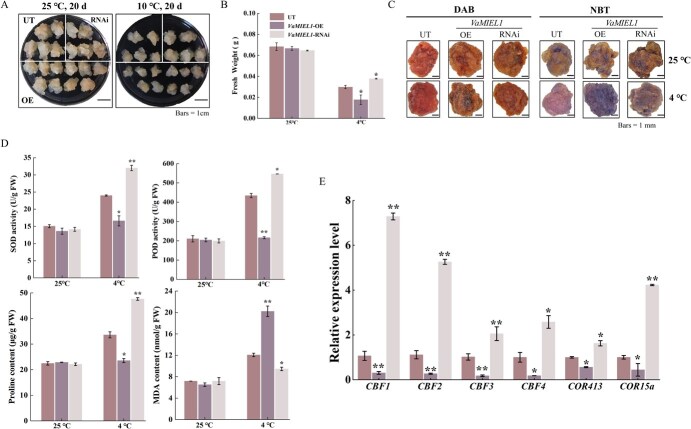
*VaMIEL1* negatively regulates cold tolerance in grapevine calli. (A) Cold stress phenotypes of UT, *VaMIEL1*-OE, and *VaMIEL1*-RNAi grapevine calli. Calli were precultured at 25°C for 10 days and then subjected to chilling stress at 10°C for 20 days. Bars = 1 cm. The images were digitally extracted for visual comparison. (B) Fresh weight of UT, *VaMIEL1*-OE, and *VaMIEL1*-RNAi grapevine calli after cold treatment. Fresh weight at 25°C was used as the control. Data represent the mean ± SD of three independent biological replicates (*n* = 6 per replicate). (C) O_2_^·−^ (Superoxide radicals) and H_2_O_2_ were detected in UT, *VaMIEL1*-OE, and *VaMIEL1*-RNAi calli via NBT and DAB staining. Calli following 3 days of cold treatment at 4°C. Bars = 1 mm. The images were digitally extracted for visual comparison. (D) Pro content, MDA content, SOD activity, and POD activity were quantified in UT, *VaMIEL1*-OE, and *VaMIEL1*-RNAi calli maintained at control conditions (25°C) or subjected to 3 days of cold stress (4°C). (E) Expression levels of cold-responsive genes in the *CBF-COR* pathway in transgenic calli under control (25°C) or following cold stress (4°C) for 3 and 12 h. Gene expression was normalized to internal reference genes. Data represent the mean ± SD of three independent experiments. Statistical significance was determined using one-way ANOVA followed by *post hoc* Tukey test (^*^P < 0.05, ^**^P < 0.01).

To evaluate the impact of *VaMIEL1* overexpression on cold tolerance in grapevine, transgenic calli overexpressing *VaMIEL1* (*VaMIEL1*-OE) and RNA interference (*VaMIEL1*-RNAi) lines were developed (Fig. S2). After 20 days of exposure to 10°C, *VaMIEL1*-OE calli exhibited pronounced signs of stress, including browning and reduced growth, compared to both untransformed (UT) and *VaMIEL1*-RNAi calli. This observation was further evidenced by a significant reduction in fresh weight under cold stress, with *VaMIEL1*-OE calli displaying markedly lower biomass accumulation compared to UT, while *VaMIEL1*-RNAi calli exhibited a notable increase. These findings suggest that VaMIEL1 negatively regulates biomass retention under low-temperature conditions (Fig. 5A and B).

Histochemical staining with NBT and DAB showed that *VaMIEL1*-OE calli accumulated significantly higher levels of ROS after 3 days of cold treatment at 4°C, suggesting heightened oxidative stress compared to UT and *VaMIEL1*-RNAi calli (Fig. 5C). Biochemical analyses corroborated these findings, showing increased MDA content and reduced Pro levels in *VaMIEL1*-OE calli, indicative of enhanced lipid peroxidation and reduced osmoprotection. Furthermore, activities of antioxidant enzymes, specifically SOD and POD, were significantly reduced in *VaMIEL1*-OE lines, indicating a weakened antioxidant defense system under cold stress (Fig. 5D). In contrast, *VaMIEL1*-RNAi calli exhibited improved biochemical markers, including higher Pro levels and enhanced SOD and POD activities, suggesting an enhanced capacity to mitigate cold-induced oxidative stress.

Furthermore, gene expression analysis of cold-regulated genes within the *CBF-COR* pathway revealed a significant downregulation in *VaMIEL1*-OE calli compared to UT controls. Notably, the expression levels of *VaCBF1* and *VaCBF3* were substantially reduced after 3 h of cold exposure at 4°C, indicating that *VaMIEL1* negatively affects the transcriptional activation of key cold-responsive genes (Fig. 5E). In contrast, *VaMIEL1*-RNAi calli exhibited a less pronounced reduction, and in some cases, even an upregulation of these genes, suggesting that silencing *VaMIEL1* may enhance cold resistance by promoting the expression of genes involved in the *CBF-COR* pathway.

### VaMIEL1 ubiquitinates and promotes the degradation of VaMYB4a through the ubiquitin–proteasome pathway

**Figure 6:**
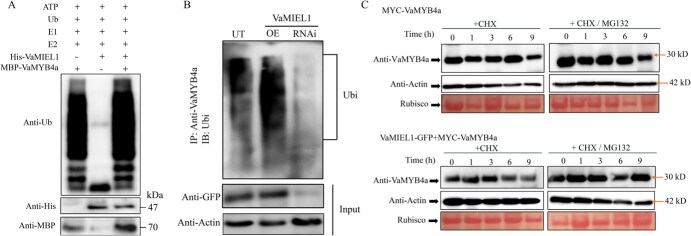
VaMIEL1 ubiquitinates and promotes the degradation of VaMYB4a. (A) *In vitro* ubiquitination of VaMYB4a by VaMIEL1. Ubiquitination assays were performed in the presence of ubiquitin (Ub), E1, E2, and His-tagged VaMIEL1, with MBP-tagged VaMYB4a as the substrate. The ubiquitination of VaMYB4a was detected using an Anti-Ub antibody. His- and MBP-tagged proteins were detected as controls. (B) *In vivo* ubiquitination of VaMYB4a by VaMIEL1. Total protein extracts from UT calli, *VaMIEL1*-OE, and *VaMIEL1*-RNAi transgenic calli were immunoprecipitated using an anti-VaMYB4a antibody. The ubiquitinated proteins were detected by WB using an Anti-Ub antibody. Anti-GFP and anti-Actin antibodies were used as controls for protein loading. (C) Degradation of VaMYB4a in cell-free degradation assays. Protein extracts from *N. benthamiana* leaves transiently expressing MYC-VaMYB4a or coexpressing MYC-VaMYB4a and VaMIEL1-GFP were treated with the protein synthesis inhibitor CHX, either alone or in combination with the proteasome inhibitor MG132. VaMYB4a protein levels were detected using an anti-VaMYB4a antibody. Actin was used as the internal reference.

*VaMIEL1*, an E3 ubiquitin ligase containing conserved domains such as a zinc ribbon finger, CHY zinc finger, and RING finger, has been implicated in the ubiquitination and subsequent degradation of VaMYB4a, based on their demonstrated physical interaction. *In vitro* assays confirmed VaMIEL1’s ability to ubiquitinate VaMYB4a. Fusion proteins His-VaMIEL1 and MBP-VaMYB4a were coincubated with ATP, ubiquitin, and the necessary E1 and E2 enzymes at 37°C for 2.5 h. WB analysis using an antiubiquitin (Anti-Ub) antibody detected ubiquitinated forms of VaMYB4a, indicating that VaMIEL1 can ubiquitinate VaMYB4a *in vitro* (Fig. 6A).

Further *in vivo* experiments corroborated this finding. Proteins extracted from UT, *VaMIEL1*-OE (overexpression), and *VaMIEL1*-RNAi grape calli were immunoprecipitated using an anti-VaMYB4a antibody, followed by WB analysis with an Anti-Ub antibody. Higher levels of VaMYB4a ubiquitination were detected in *VaMIEL1*-OE calli compared to UT and RNAi lines, suggesting that VaMIEL1 mediates VaMYB4a ubiquitination *in vivo* (Fig. 6B).

To further assess VaMIEL1’s role in the degradation of VaMYB4a, cell-free degradation assays were performed using *Nicotiana benthamiana* leaves transiently expressing MYC-VaMYB4a, with or without VaMIEL1-GFP coexpression. MYC-VaMYB4a protein levels were monitored over time in the presence of the protein synthesis inhibitor cycloheximide (CHX), with or without the proteasome inhibitor MG132. Coexpression of VaMIEL1-GFP accelerated the degradation of MYC-VaMYB4a, reducing its protein levels significantly. However, treatment with MG132 partially rescued VaMYB4a from degradation, indicating that VaMIEL1 promotes VaMYB4a degradation via the ubiquitin–proteasome pathway (Fig. 6C).

### *VaMIEL1* and *VaMYB4a* co-regulate cold tolerance in grapevine by modulating the *CBF-COR* signaling pathway

**Figure 7:**
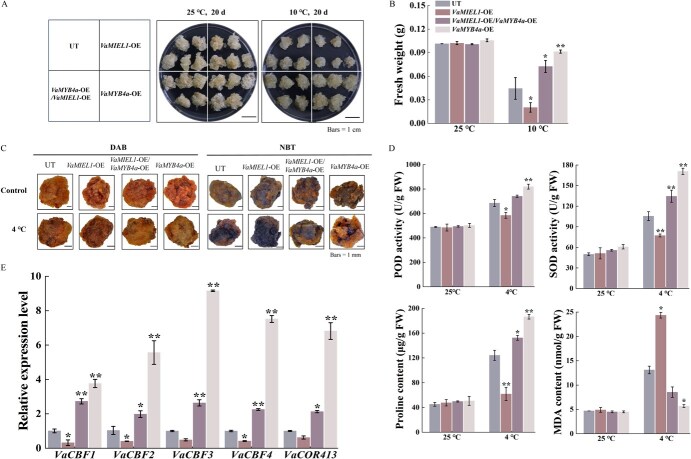
Enhanced cold tolerance in grapevine calli mediated by *VaMIEL1* and *VaMYB4a* interactions through the *CBF-COR* signaling pathway. (A) Cold stress phenotypes of grapevine calli overexpressing *VaMIEL1*, *VaMYB4a*, or coexpressing both. Calli were cultured at 25°C for 10 days prior to exposure to chilling stress at 10°C for 20 days. Scale bars = 1 cm. (B) Fresh weight of UT, *VaMIEL1*-OE, *VaMYB4a*-OE, and coexpressed *VaMIEL1*-OE/*VaMYB4a*-OE grapevine calli after cold treatment at 10°C. (C) Detection of O_2_^·-^ and H_2_O_2_ in UT, *VaMIEL1*-OE, *VaMYB4a*-OE, and *VaMIEL1*-OE/*VaMYB4a*-OE calli using NBT and DAB staining. Calli were cold treated at 4°C for 3 days. Bars = 1 mm. The images were digitally extracted for visual comparison. (D) Quantification of Pro content, MDA content, SOD activity, and POD activity in transgenic calli maintained under control conditions (25°C) or exposed to cold stress (4°C) for 3 days. (E) Expression levels of cold-regulated genes in the *CBF-COR* pathway in transgenic calli under control (25°C) or after exposure to cold stress (4°C) for 3 and 12 h. Data represent the mean ± SD of three independent experiments. Statistical significance was determined using one-way ANOVA followed by *post hoc* Tukey test (^*^P < 0.05, ^**^P < 0.01).

To clarify the interactive effects of *VaMIEL1* and *VaMYB4a* on cold tolerance in grapevine, calli overexpressing *VaMYB4a* (Fig. S3) and coexpressing *VaMYB4a* with *VaMIEL1* (Fig. S4) were developed. After cold treatment, calli coexpressing *VaMYB4a* and *VaMIEL1* exhibited a fresh weight increase of 0.028 g compared to those overexpressing only *VaMIEL1*. However, compared to calli solely overexpressing *VaMYB4a*, the coexpressing calli demonstrated reduced vigor and diminished cold tolerance, as evidenced by a 0.02 g decrease in fresh weight gain under cold conditions (Fig. 7A and B).

Chemical staining with DAB and NBT under low-temperature stress revealed lighter staining in both *VaMYB4a*-OE and coexpressed calli, indicating lower levels of ROS, while *VaMIEL1*-OE calli exhibited more intense and variable staining, suggesting higher ROS accumulation (Fig. 7C). Biochemical analyses under cold stress showed increased POD and SOD activities in coexpressed calli, with levels rising by 55.5 and 29 U/g FW, respectively, compared to UT. However, relative to *VaMYB4a*-OE calli, POD and SOD activities in coexpressed calli were reduced by 79 and 36 U/g FW, respectively.

Additionally, under 4°C cold stress, coexpressed calli exhibited a significant increase in Pro content (28.3 μg/g FW) and a marked decrease in MDA content (4.6 nmol/g FW) compared to the control condition at 25°C. In contrast, relative to *VaMYB4a*-OE calli, coexpressed tissues displayed a reduction in Pro content by 34 μg/g FW and an increase in MDA content by 3.1 nmol/g FW (Fig. 7D). These results suggest that while the interaction between *VaMIEL1* and *VaMYB4a* enhances overall cold resistance, it also mitigates the cold tolerance specifically mediated by *VaMYB4a* alone.

To further investigate the impact of coexpression of *VaMIEL1* and *VaMYB4a* on the CBF-COR signaling pathway, we analyzed the expression patterns of *CBF* and *COR* genes in coexpressed grapevine calli under cold stress. Upon exposure to 4°C, the expression levels of cold-responsive genes particularly *VaCBF1* and *VaCBF3* were significantly upregulated. In coexpressed calli, the expression of *VaCBF1,* and *VaCBF3* increased by ~1.7- and 1.6-fold, respectively, compared to UT, whereas in *VaMYB4a*-OE calli, these levels rose by 2.7- and 8.1-fold, respectively (Fig. 7E). These findings suggest that *VaMYB4a* counteracts the reduced cold tolerance observed in *VaMIEL1*-OE calli, and the interplay between *VaMIEL1* and *VaMYB4a* modulates cold tolerance through the *CBF-COR* signaling pathway.

## Discussion

Our study investigates the role of *VaMIEL1*, a RING-type E3 ubiquitin ligase, in regulating cold tolerance in *V. amurensis* through its interaction with the transcription factor VaMYB4a. Our central hypothesis posited that VaMIEL1 functions as a negative regulator of cold tolerance by promoting the ubiquitination and degradation of VaMYB4a, thereby modulating cold-responsive gene expression. Through *in vivo* and *in vitro* experiments, we demonstrated that overexpression of VaMIEL1 impairs cold tolerance by enhancing VaMYB4a degradation, leading to a downregulation of key cold-responsive genes in the *CBF-COR* pathway and an unexpected increase in oxidative stress. These findings suggest a dual role for *VaMIEL1* in both transcriptional regulation and redox homeostasis, offering new insights into the complex coordination of cold stress responses.

Our findings align with prior studies on the role of E3 ubiquitin ligases in cold stress responses. Similar to HOS1 in *Arabidopsis*, which targets the transcription factor ICE1 for degradation, thus reducing cold tolerance [15, 16], we found that VaMIEL1 targets VaMYB4a, a transcriptional activator of cold-responsive genes, for proteasomal degradation. This confirms a conserved mechanism across species, wherein E3 ligases regulate the stability of cold-responsive transcription factors to modulate stress tolerance [23]. The apple U-box-type E3 ubiquitin ligase MdPUB23 diminishes the cold-stress regulatory protein MdICE1 for degradation [17]. PUB25 and PUB26 modulate ICE1 stability dynamically through differential ubiquitination during cold stress [24]. Moreover, our results are consistent with those of An *et al.* [25], who showed that the E3 ligase MdMIEL1 in apple reduces cold tolerance by targeting MYB transcription factors for degradation. By uncovering a similar mechanism in *V. amurensis*, our study extends the role of E3 ligases beyond model species, emphasizing their importance in perennial crops like grapevine.

However, we observed an additional layer of complexity that sets our study apart. While previous research focused primarily on transcription factor degradation, we found that *VaMIEL1* overexpression significantly increases ROS accumulation (Fig. 4E), suggesting a more intricate interplay between E3 ligases and oxidative stress regulation during cold stress. ROS accumulation is well documented as a byproduct of cold stress [26–29]; however, the impairment of antioxidant defenses—marked by reduced SOD and POD activities in *VaMIEL1*-OE lines (Fig. 4F)—indicates that *VaMIEL1* has a broader regulatory role that includes modulating redox homeostasis. This finding contrasts with previous studies on E3 ligases where the focus has been predominantly on transcription factor degradation, without explicit links to ROS regulation. The implication is that *VaMIEL1* may be influencing multiple pathways simultaneously, integrating transcriptional control with redox regulation [30].

**Figure 8:**
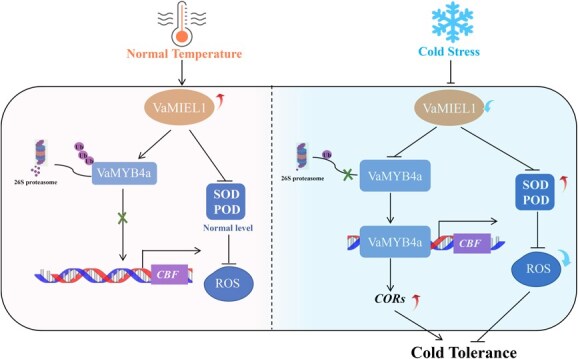
Working model of the VaMIEL1-VaMYB4a module in response to cold stress in grapevine. Under normal temperature conditions (left panel), *VaMIEL1* is highly expressed and promotes the degradation of VaMYB4a via the 26S proteasome pathway, leading to a suppression of CBF transcription. Consequently, the antioxidant enzyme levels (SOD, POD) remain at normal levels, and ROS are maintained at a steady state. Cold-responsive (*COR*) genes are not activated, and cold tolerance is not induced. Under cold stress conditions (right panel), *VaMIEL1* expression is downregulated, reducing VaMYB4a ubiquitination and allowing its accumulation. The stabilized VaMYB4a binds to the *CBF* promoter, activating its expression. Increased *CBF* levels lead to the induction of downstream COR genes, enhancing cold tolerance. Additionally, the upregulation of SOD and POD contributes to ROS scavenging, further improving stress adaptation. The black arrows represent inducible expression, while flat-ended lines indicate inhibition. Red and blue arrows denote changes in enzyme activity and expression levels, respectively. The red ‘X’ indicates inhibited regulation, and Ub denotes ubiquitination modifications.

One particularly unexpected finding was the extent of oxidative stress in *VaMIEL1*-OE lines. Contrary to expectations and earlier reports emphasizing the transcriptional regulation role of E3 ligases, we observed a significant increase in ROS levels, along with decreased activities of SOD and POD (Fig. 4E, 4F). This suggests that *VaMIEL1* disrupts the plant's ability to maintain redox homeostasis, exacerbating oxidative damage under cold stress conditions. This dual role of *VaMIEL1*—modulating both gene expression and ROS detoxification—offers a new perspective on how plants coordinate responses to cold stress at multiple cellular levels. Specifically, VaMIEL1 appears to act as a critical regulator that integrates transcriptional and oxidative stress signals, providing a potential mechanism for how plants fine-tune their stress responses. This novel role of *VaMIEL1* in ROS regulation could have broader implications for understanding how plants adapt to cold environments, and future studies should explore whether other E3 ligases exhibit similar dual regulatory functions. These findings expand our overall understanding of the UPS and its critical function in plant stress responses. While the UPS has traditionally been viewed as a selective degradation system for proteins, particularly transcription factors [31–34], our results suggest that E3 ligases like *VaMIEL1* play a more complex role that extends beyond transcriptional regulation. The significant increase in ROS levels observed in *VaMIEL1*-OE lines (Fig. 7) indicates that ubiquitination may also regulate oxidative stress responses, highlighting a novel regulatory role for E3 ligases in modulating cellular redox balance. Our study challenges the prevailing view that E3 ligases solely mediate transcription factor turnover [35, 36], proposing that they also play an integral role in redox homeostasis. By regulating both gene expression and ROS detoxification, *VaMIEL1* exemplifies how E3 ligases may operate at the intersection of multiple stress signaling pathways, fine-tuning the plant’s response to environmental stressors (Fig. 8). This revised model has broad implications for our understanding of plant stress tolerance, suggesting that future research should examine the nontranscriptional roles of E3 ligases in stress adaptation.

Despite the valuable insights provided by this study, several limitations must be acknowledged. A key limitation of this study lies in the use of *A. thaliana* as a heterologous system for the overexpression and T-DNA insertion of *miel1* mutant. While *Arabidopsis* serves as a valuable model for functional analysis, the physiological responses to cold stress in *Arabidopsis* may not fully mirror those of *V. amurensis*, given their distinct native responses to low-temperature environments. The *miel1* mutant in *Arabidopsis* exhibited increased sensitivity to cold, as evidenced by heightened ROS accumulation and altered *CBF-COR* pathway gene expression (Fig. 4E, 4F, 4G). However, these responses may differ from those in *V. amurensis*, where native cold tolerance mechanisms, including antioxidant regulation, may operate differently (Fig. 5D). These species-specific differences could limit the direct generalizability of the findings from *Arabidopsis* to grapevine [37–39]. Therefore, future studies should validate these results in *V. amurensis* or other grapevine species under natural cold stress conditions to fully assess the physiological relevance of VaMIEL1 in its native context. Additionally, our analysis of ROS dynamics focused primarily on SOD and POD activities. A broader investigation into other antioxidant enzymes, as well as non-enzymatic ROS scavengers, would provide a more comprehensive understanding of how VaMIEL1 modulates oxidative stress under cold conditions [26, 40–42]. Further studies could explore whether VaMIEL1 influences other cellular processes that contribute to redox balance, such as the metabolism of ascorbate and glutathione, which are known to play key roles in stress tolerance.

In conclusion, our study identifies *VaMIEL1* as a central negative regulator of cold tolerance in *V. amurensis*, functioning through the targeted degradation of VaMYB4a and disruption of ROS homeostasis. By demonstrating VaMIEL1’s dual role in both transcriptional regulation and oxidative stress management, our findings challenge current models of plant stress tolerance and highlight the potential for E3 ligases to act as integrators of multiple stress signaling pathways. These insights open up new avenues for improving cold tolerance in crops through molecular breeding or genetic engineering, ultimately enhancing the resilience of economically important species in the face of increasingly variable climate conditions.

## Materials and methods

### Plant materials and growth conditions

The Chinese wild *V. amurensis* Rupr. ‘Zuoshan-1’ was cultivated under controlled greenhouse conditions at Ningxia University, Yinchuan, Ningxia Hui Autonomous Region, China (38°30′13.44″N; 106°08′08.04″E). For tissue-specific expression analysis, plant materials including roots, leaves, shoots, and tendrils were harvested from healthy plants grown in pots containing a 3:1:1 mixture of vegetative soil, perlite, and vermiculite. Cold stress treatments were applied to the seedlings in a growth chamber set at −2°C, following the protocol described [43]. Leaf samples were flash-frozen promptly in liquid nitrogen following collection and stored at −80°C for subsequent use.

Additionally, *A. thaliana* ecotype Columbia-0 (Col-0) seeds were sourced from the Nottingham Arabidopsis Stock Centre (NASC). Seedlings were grown on half-strength MS medium under long-day conditions (16 h light/8 h dark) at 22°C in a growth chamber. The seedlings were used for cold tolerance assays and transgenic experiments as described below.

### Yeast two-hybrid analysis

The interaction between VaMIEL1 and VaMYB4a was tested using the Matchmaker™ Gold Yeast Two-Hybrid System (Clontech). Full-length VaMIEL1 was cloned into the pGADT7 vector (prey), while full-length VaMYB4a and its truncated forms (VaMYB4a^1-112^ and VaMYB4a^113-252^) were cloned into the pGBKT7 vector (bait). Primers used for vector construction are listed in Table S2. Cotransformed *Saccharomyces cerevisiae* strain Y2HGold cells were initially selected on SD/−Leu/−Trp media, followed by interaction screening on SD/−Leu/−Trp/–His/−Ade media supplemented with X-α-Gal. Positive interactions were indicated by colony growth and blue coloration. Yeast growth was monitored after incubation at 30°C for 3 days, as previously described [44]. Empty pGBKT7 and pGADT7 vectors were used as negative controls, while p53-T and Lamin-C constructs served as positive and negative interaction controls, respectively. All Y2H assays were repeated three times independently to confirm reproducibility.

### Bimolecular fluorescence complementation assay

BiFC assays were conducted to confirm the interaction between VaMIEL1 and VaMYB4a *in planta*. *VaMIEL1* was fused to the N-terminal half of YFP (nYFP), and V*aMYB4a* to the C-terminal half (cYFP). The isolation and transformation of *A. thaliana* protoplasts were conducted using the *Agrobacterium tumefaciens* strain GV3101(pSoup), following previously established protocols [45, 46]. After 48 h of incubation at 22°C, fluorescence signals resulting from YFP reconstitution were visualized using a Leica TCS SP5 confocal laser scanning microscope, as described [47]. Negative controls included protoplasts coexpressing unfused nYFP and cYFP vectors. To assess the effect of proteasomal inhibition on the interaction, protoplasts were treated with 50 μM MG132. BiFC assays were performed in triplicate with consistent results.

### Co-immunoprecipitation assays

To investigate the role of *VaMIEL1*, both *VaMIEL1*-GFP and *VaMIEL1*-RNAi constructs were transiently expressed in ‘Chardonnay’ grapevine calli. Following transformation, calli were treated with 100 μM MG132 for 8 h to inhibit proteasomal degradation. Total protein was extracted using a buffer containing 25 mM Tris-HCl (pH 7.5), 10 mM NaCl, 10 mM MgCl₂, 5 mM dithiothreitol (DTT), 10 mM ATP, and 4 mM phenylmethylsulfonyl fluoride (PMSF). Extracted proteins were first incubated with beads for 30 min to remove nonspecific proteins, followed by immunoprecipitation with a rabbit anti-*VaMYB4a* antibody (ABclonal, Wuhan, China). The mixture was incubated at 4°C for 4 h or overnight. Protein A/G Plus beads were then added, and the mixture was incubated for an additional 3 h at 4°C. After incubation, beads were washed five times with extraction buffer to eliminate nonspecific interactions, and immunoprecipitated complexes were resuspended in protein loading buffer. Samples were separated on 10% SDS-PAGE and analyzed by immunoblotting with anti-GFP serum to detect target proteins, as previously described [48].

### Sequence analysis of *VaMIEL1*

The *VaMIEL1* gene was amplified by PCR from cDNA extracted from ‘Zuoshan-1’ leaf tissue. The ORF of VaMIEL1 was analyzed using NCBI's ORF Finder tool (https://www.ncbi.nlm.nih.gov/orffinder/). Chromosomal location and exon–intron structure were visualized with the Exon–Intron Graphic Maker (http://wormweb.org/exonintron). Conserved protein domains were predicted through the SMART tool (http://smart.embl-heidelberg.de/), and sequence alignments were conducted using GeneDoc. Phylogenetic relationships were inferred in MEGA7 (version 7.0.26) using the neighbor-joining method, with 1000 bootstrap replicates to evaluate clade stability.

### Reverse-transcription quantitative PCR analysis

Total RNA was isolated using the Plant RNA Kit (OMEGA, Shanghai, China) and subsequently reverse-transcribed with the PrimeScript™ RT Reagent Kit and gDNA Eraser (TaKaRa, Shiga, Japan), following the protocols provided by the manufacturers. RT-qPCR was performed using the 2 × SYBR Green qPCR Mix (Aidlab, Beijing, China) on a qTOWER 2.2 qRT-PCR Thermal Cycler (Analytik Jena AG, Thuringia, Germany). The RT-qPCR cycle settings included an initial denaturation at 94°C for 2–3 min, followed by 40 cycles at 94°C for 10 s and 60°C for 30 s. Primer sequences are detailed in Table S2. Gene expression data were normalized to *VvActin1* (GenBank accession number XM_002282480), with relative expression levels calculated via the 2^-ΔΔCT^ method [49]. Each experiment was conducted in triplicate.

### GUS staining and fluorometric GUS activity assay

The full-length *VaMIEL1* promoter was inserted into the pCAMBIA2301::GUS vector and subsequently introduced into ‘Chardonnay’ grape calli to evaluate cold tolerance at 4°C. GUS staining was carried out using a GUS staining kit (SL7160, Coolaber, China), with samples incubated at 37°C for 6 h, followed by decolorization in 70% ethanol, as previously described [50]. For quantitative assessment of GUS activity, transformed calli were ground in liquid nitrogen, and total protein was extracted using a suitable buffer. Enzyme reactions were conducted with 4-Methylumbelliferyl beta-D-glucuronide (4-MUG) (Sigma-Aldrich, St. Louis, MO) as the substrate, with 1 mg of total protein incubated at 37°C. GUS activity was quantified using a Versa Fluor spectrofluorometer (Bio-Rad, Shanghai, China) with an excitation wavelength of 365 nm and emission at 450 nm, following the established method [51]. Each measurement was performed in triplicate.

### *Arabidopsis* transformation and mutant verification

*Arabidopsis thaliana* were transformed via the floral dip method [52]. Transgenic lines were selected on MS agar medium containing 50 μg/ml kanamycin, and T3 generation plants were used for subsequent experiments. The *miel1* mutant (SALK_087883) was sourced from the SALK collection at the *Arabidopsis* Information Resource (TAIR), with the T-DNA insertion confirmed by PCR using primers LBb1.3, LP1, and RP1. Transcript levels in the transgenic plants were assessed by RT-qPCR, and protein expression was examined through immunoblot analysis. Primer sequences are detailed in Table S2.

### Generation of transiently transformed and stably transformed grapevine calli

To generate overexpression vectors for *VaMIEL1* in grapevine calli, the coding sequences of *VaMIEL1* were cloned into the pGWC-Fan vector using the Gateway cloning system. Specific primers, designed based on the *VaMIEL1* gene sequence (Table S2), were used to amplify the target fragment from the AD-*MIEL1* yeast expression vector. *Ahd* I was chosen as the restriction enzyme for site-specific cleavage, enabling the insertion of the PCR product into the pGWC-Fan vector. The resulting plasmids were subjected to LR recombination with the pHZM03 vector to generate the final expression constructs, which were introduced into *A. tumefaciens* strain GV3101 (pSoup) as described previously [53].

Stable transformation of grapevine calli and identification of positive transgenic lines were performed using protocols established by our group [53, 54]. Transformed calli were subjected to selection over a 40-day period on MS medium supplemented with 2.0 mg·l^−1^ 6-benzylaminopurine (6-BA), 0.6 mg·l^−1^ MEL5, 30 g·l^−1^ sucrose, 3 g·l^−1^ Phytagel, 1 g·l^−1^ activated carbon, 300 mg·l^−1^ carbenicillin, and 75 mg·l^−1^ kanamycin.

### *In vitro* and *in vivo* ubiquitination

For the *in vitro* ubiquitination assay, the coding sequence of full-length *VaMIEL1* was amplified by PCR and cloned into the pET32a-6 × His vector (GE Healthcare, Fairfield, CT, USA). Specific primers, designed based on the *VaMIEL1* gene sequence (Table S2), were used to amplify the target fragment from the AD-*MIEL1* plasmid. The PCR product was subsequently double-digested with *EcoR* I and *Xho* I and ligated into the pET32a-6 × His vector. His-tagged *VaMIEL1* fusion protein was expressed in *Escherichia coli* Rosetta strain and purified using the HIS Tagged Protein Purification Kit (P2012, Solarbio, China). The ubiquitination reaction mixture contained MBP-*VaMYB4a*, His-*VaMIEL1*, ATP (SL1262, Coolaber, China), ubiquitin (UB-102H-1 M, Youbi, China), UBE1 (UBE-024, Youbi, China), and UBE2D2 (UBE-622, Youbi, China), and was incubated at 37°C for 2.5 h. Ubiquitination was assessed by WB analysis using an Anti-Ub monoclonal antibody, following established protocols [55].

For the *in vivo* ubiquitination assay, total proteins were isolated from *VaMIEL1*-OE, *VaMIEL1*-RNAi transgenic plants, and UT calli using an extraction buffer containing 25 mM Tris-HCl (pH 7.5), 10 mM NaCl, 10 mM MgCl₂, 5 mM DTT, 10 mM ATP, and 4 mM PMSF. The extracted proteins were immunoprecipitated with an anti-*VaMYB4a* polyclonal antibody (ABclonal, Wuhan, China) and subsequently subjected to immunoblotting with an Anti-Ub antibody (Cell Signaling Technology, USA) [56]. A separate immunoblot was performed using an anti-Actin antibody as a loading control.

### Cell-free degradation analyses

Cell-free degradation assays were conducted using 4-week-old *N. benthamiana* plants [57]. *VaMIEL1*-GFP and MYC-*VaMYB4a* constructs were introduced into *N. benthamiana* leaves via agroinfiltration. Following infiltration, leaves were collected, and total proteins were extracted with buffer containing 50 mM Tris-HCl (pH 8.0), 0.5 mM sucrose, 1 mM MgCl₂, 10 mM EDTA, and 5 mM DTT, supplemented with freshly added protease inhibitor cocktail (Complete Mini tablets, Roche, Shanghai, China). Extracted proteins were then incubated with 50 μM CHX at 29°C for varying time intervals (0–100 min) in the presence or absence of 50 μM MG132, a proteasome inhibitor. After incubation, protein was separated by SDS-PAGE, and VaMYB4a levels were analyzed via WB. Assays were repeated in three independent experiments.

### Evaluation of cold tolerance in transgenic ***Arabidopsis*** and grapevine calli

Cold tolerance was evaluated in WT and transgenic *A. thaliana* lines grown on half-strength MS medium, subjected to either cold acclimation (CA) or non-cold acclimation (NA) treatments. For CA treatment, plants were preconditioned at 4°C for 4 days, then exposed to −7°C for 1.5 h. In the NA treatment, plants were exposed directly to −5°C for 1.5 h without prior cold acclimation. Following freezing treatments, plants were returned to standard growth conditions for a 2-day recovery, after which survival rates were determined by counting seedlings that remained viable postfreezing [58]. Six biological replicates were used for each treatment. Photographs were taken before and after freezing treatment and recovery to document plant conditions. Leaf samples were collected to measure ion leakage, indicating cellular damage caused by freezing stress.

*Vitis vinifera* ‘Chardonnay’ grapevine calli were first transformed with *VaMIEL1*-GFP, followed by a second transformation with MYC-*VaMYB4a*. The transformed calli were selected on MEL5 medium supplemented with 30 mg·l^−1^ kanamycin and 5 mg·l^−1^ hygromycin, cultured in darkness for 30 days to facilitate transgenic calli development [54]. To maintain culture stability, calli were subcultured biweekly on the same selective medium.

*In situ* detection of ROS involved DAB staining for H₂O₂ visualization and nitroblue tetrazolium (NBT) staining for superoxide anion (O_2_^·−^) detection. SOD and POD enzyme activities, along with MDA and Pro concentrations, were quantified using assay kits specific for each parameter (SOD, A001-3-2; POD, A084-3-1; MDA, A003-1-1; Pro, A107-1-1) from Nanjing Jiancheng Bioengineering Institute, Nanjing, China. For analysis of cold stress-responsive gene expression, samples were collected after 3 and 12 h at 4°C [59]. RT-qPCR was conducted using *AtActin1* (GenBank accession number AT2G37620) as a reference gene, with UT samples serving as negative controls. Primers for RT-qPCR are listed in Table S2.

### Statistical analysis

Statistical analyses were performed with SPSS software (version 24.0, USA). Results are expressed as mean ± SD. Analysis of variance (ANOVA), followed by Tukey’s *post hoc* test, was used to assess statistical significance among multiple groups, while comparisons between two groups were analyzed using an independent *t*-test. Significance levels are denoted as ^*^P < 0.05, ^**^P < 0.01, and ^***^P < 0.001.

## Accession number

Sequence data referenced in this article are available in the National Center for Biotechnology Information database under the following accession numbers: VaMIEL1 (PP471211). Supplementary data

## Acknowledgements

The gift of the Arabidopsis mutant miel1 (SALK_087883) from Zheng Yuan’s team at the State Key Laboratory of Crop Adaptation and Improvement, School of Life Sciences, Henan University, is greatly appreciated. This work is supported by Ningxia Hui Autonomous Region Key R&D Program (Grant no. 2023BCF01003), National Natural Science Foundation of China (Grant no. 32472711 and 32060672), and Agricultural Breeding Project of Ningxia Hui Autonomous Region (Grant no. NXNYYZ202101).

## Supplementary Material

Web_Material_uhaf093

## Data Availability

The authors confirm that the data supporting the findings of this study are available within the article.
