# Supplementary material for: VaMIEL1-mediated ubiquitination of VaMYB4a orchestrates cold tolerance through integrated transcriptional and oxidative stress pathways in grapevine
Source: Hortic Res. 2025 Mar 22;12(7):uhaf093. doi: 10.1093/hr/uhaf093 (PMC12087447; doi:10.1093/hr/uhaf093)
Supplement: Web_Material_uhaf093 [file web_material_uhaf093.zip › Supplementary_Figure_uhaf093.docx]

**Supplementary Figure**


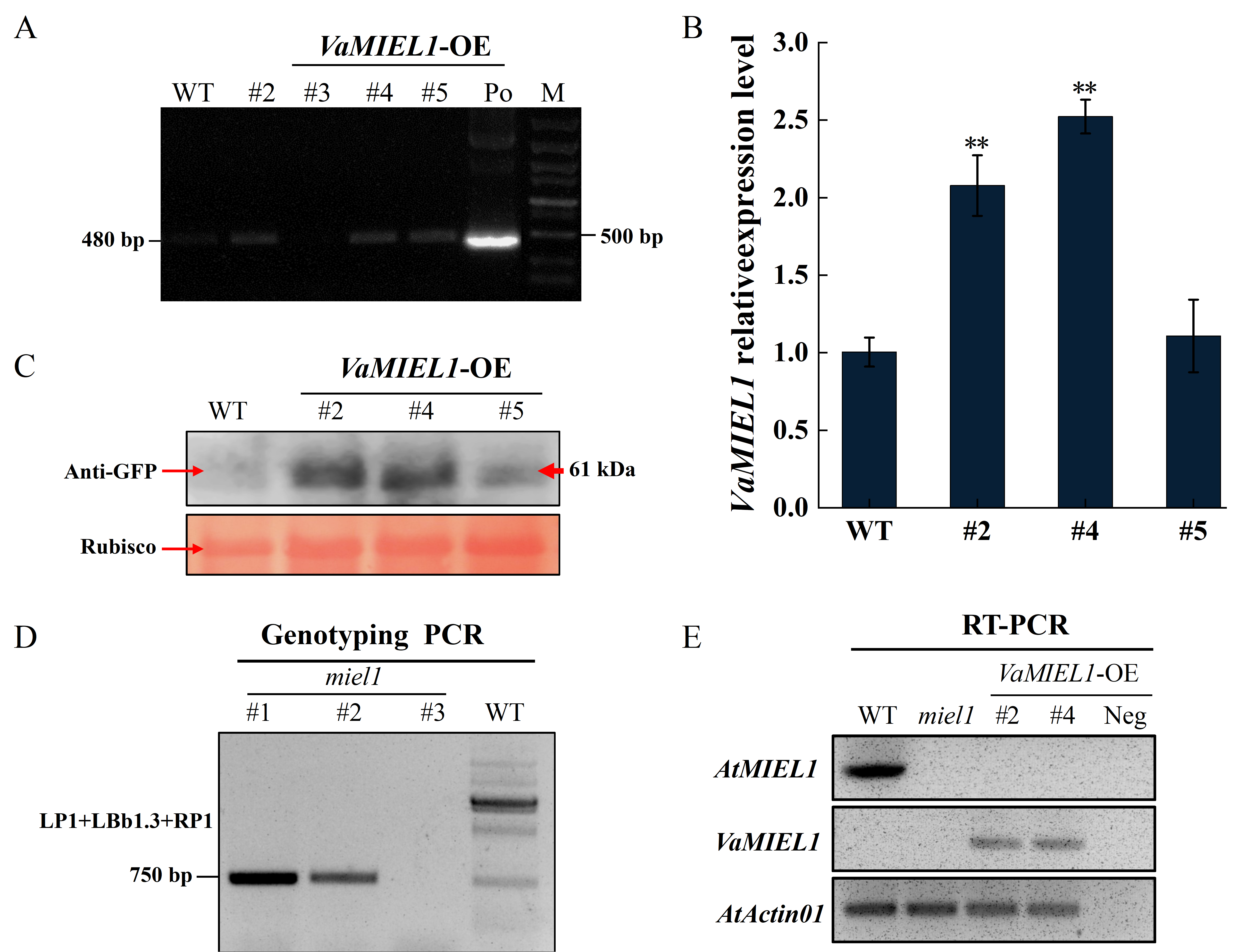


**Figure S1. Identification and characterization of VaMIEL1-OE transgenic Arabidopsis and miel1 mutants.**
**(A)** PCR confirmation of VaMIEL1-OE (VaMIEL1 overexpression) transgenic Arabidopsis thaliana lines. The presence of the transgene was verified in independent transgenic lines (#2, #3, #4 and #5) and compared to wild-type (WT) plants. M: DNA marker, PO: positive control. **(B)** Quantification of VaMIEL1 expression levels in VaMIEL1-OE transgenic Arabidopsis lines (#2, #4 and #5) relative to WT, as determined by RT-qPCR. ***p* < 0.01 between WT and transgenic lines, as determined by one-way ANOVA. **(C)** Immunoblot analysis of VaMIEL1-OE transgenic Arabidopsis lines (#2, #4, #5) using anti-GFP antibody to detect GFP-tagged VaMIEL1. Rubisco staining served as a loading control. **(D)** PCR-based genotyping of miel1 homozygous mutant lines. PCR was performed using LP1, LBb1.3 and RP1 primers, with WT as a control.
**(E)** RT-qPCR analysis of AtMIEL1 and VaMIEL1 expression in WT plants, miel1 mutants, and VaMIEL1-OE transgenic lines (#2, #4). AtActin1 was used as an internal control. Neg: negative control (no template).


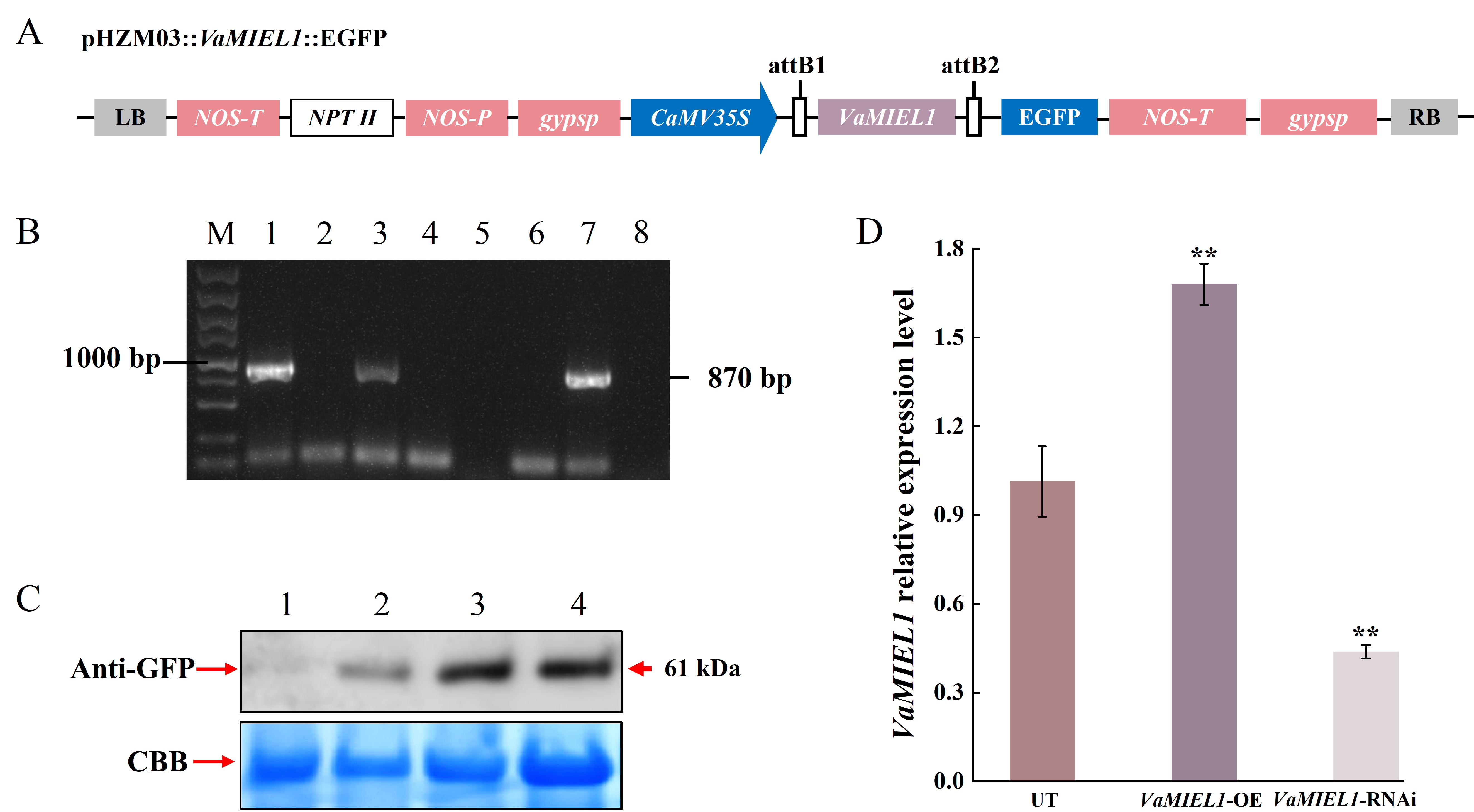


**Figure S2. Identification of VaMIEL1 transgenic grapevine calli.**

(A) Schematic map of the *pHZM03::VaMIEL1::EGFP* vector used for generating *VaMIEL1*-EGFP fusion protein expression. (B) PCR-based identification of *VaMIEL1* in UT and *VaMIEL1*-transformed grapevine calli. Amplification of *VaMIEL1* in positive transgenic calli yields an 870 bp product. Plasmid pHZM03-*VaMIEL1* was used as a positive control (Po). M: DNA Marker 5000; lane 1: Po; lanes 2-7: representative *VaMIEL1*-overexpression (OE) calli; lane 8: UT negative control (Ne). (C) Western blot detection of VaMIEL1 protein expression in representative transformed grapevine calli lines. Calli with good protein expression were selected for RT-qPCR analysis. Lane 1: UT; lanes; 2-4: representative *VaMIEL1*-OE calli. Coomassie Brilliant Blue (CBB) staining was used as a loading control. (D) RT-qPCR analysis of relative expression levels of *VaMIEL1* in UT, *VaMIEL1*-OE, and *VaMIEL1*-RNAi grapevine calli. Data represent the mean ± SD of three independent experiments. Asterisks indicate statistically significant differences compared to the UT control (**p* < 0.05, ***p* < 0.01; one-way ANOVA with post-hoc Tukey test).


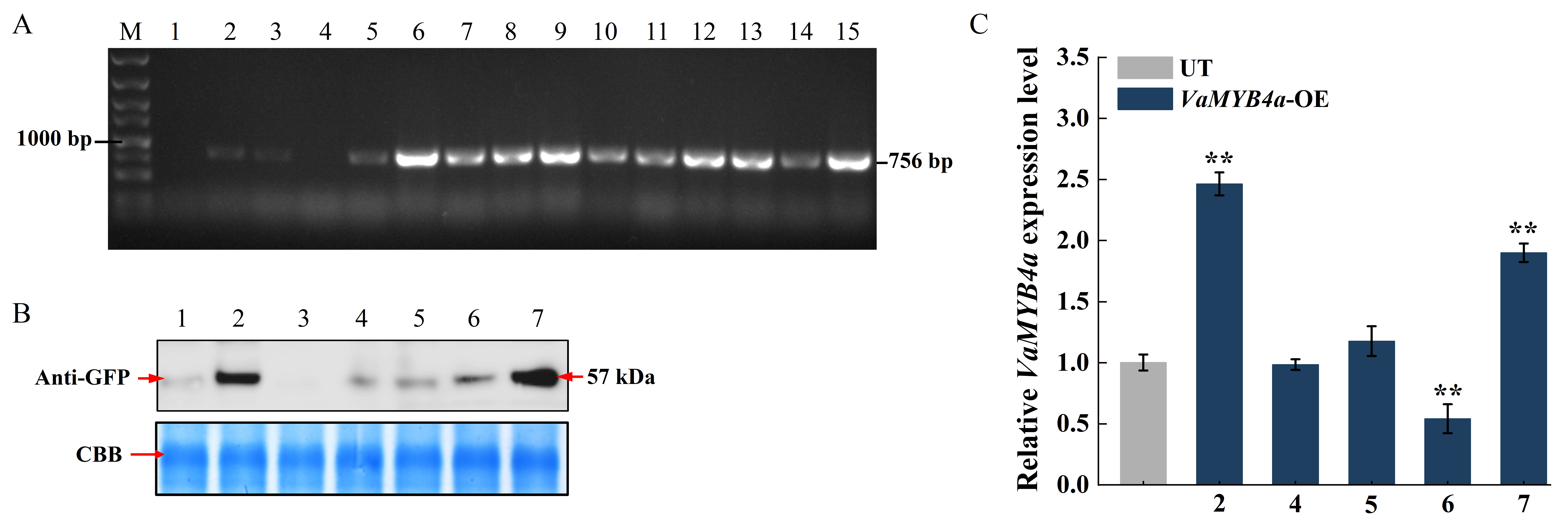


**Figure S3. Identification of VaMYB4a overexpressing grapevine calli.**
**(A)** PCR-based confirmation of VaMYB4a overexpression in transformed grape calli. A 756 bp product indicates successful amplification of VaMYB4a. Plasmid pHZM03-VaMYB4a was used as a positive control (Po), and UT calli served as a negative control (Ne). M: DNA marker (5000 bp); lane 1: UT; lanes 2-14: representative VaMYB4a-OE (overexpression) calli; lane 15: Po. **(B)** Western blot detection of VaMYB4a protein expression in transformed lines. Lane 1: UT; lanes 2-7: representative VaMYB4a-OE calli. Coomassie Brilliant Blue (CBB) staining was used as a loading control. **(C)** RT-qPCR analysis of endogenous VaMYB4a expression levels in UT and VaMYB4a-OE calli. For gene expression analysis, lanes 2, 4, 5, 6 and 7 proteins in (B) were selected. Data represent the mean ± SD of three independent experiments. Asterisks indicate statistically significant differences compared to the UT control (**p* < 0.05, ***p* < 0.01; one-way ANOVA with post-hoc Tukey test).


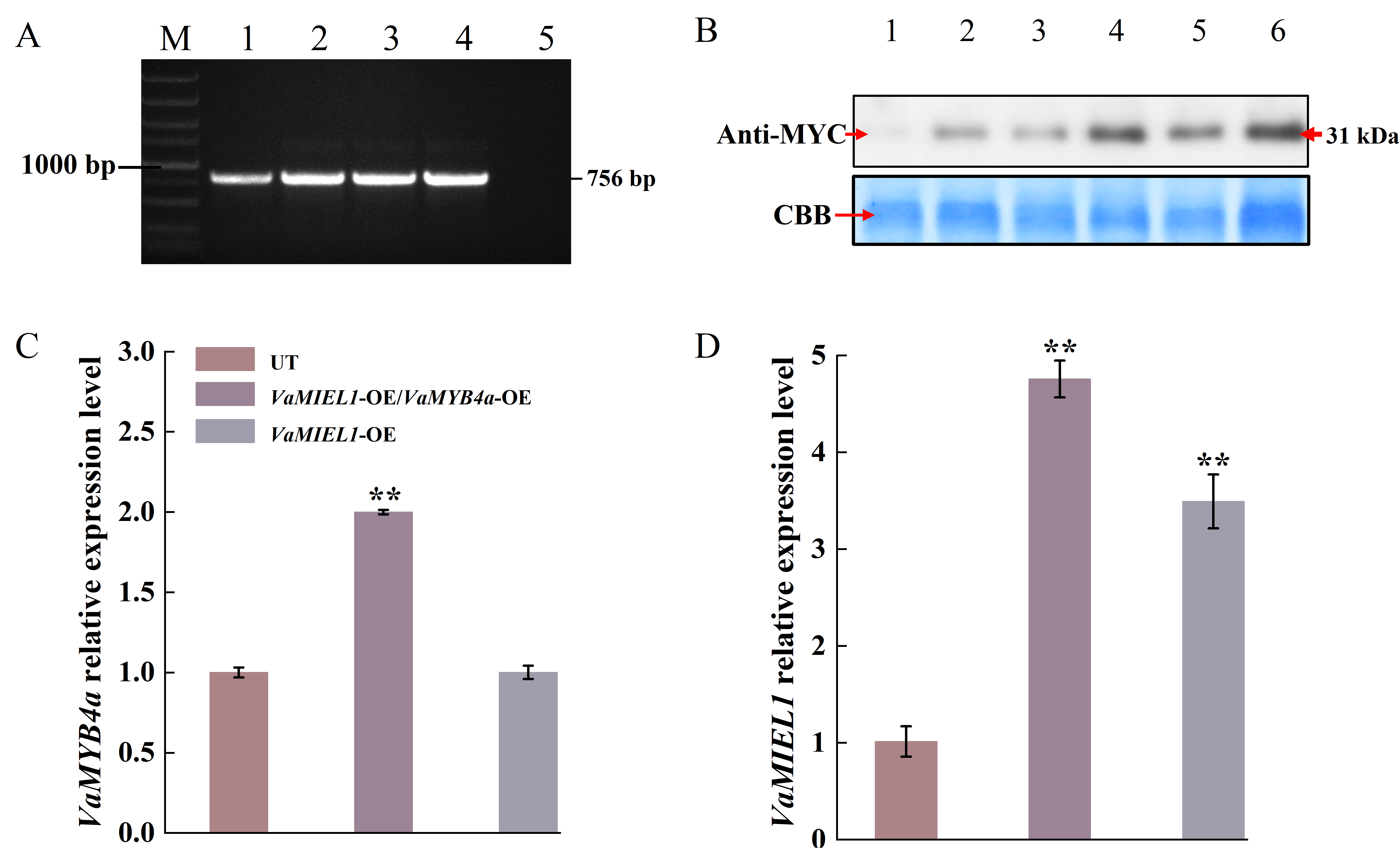


**Figure S4. Identification of VaMIEL1 and VaMYB4a co-expressed grapevine calli.**
**(A)** PCR-based confirmation of VaMYB4a expression in UT and VaMIEL1-transgenic grapevine calli. Amplification of VaMYB4a in positive calli yields a 756 bp product. Plasmid pCAMBIA1307-VaMYB4a was used as a positive control (Po). Lanes 1-3: VaMIEL1 and VaMYB4a co-expressed calli; lane 4: Po; lane 5: UT negative control (Ne). **(B)** Western blot analysis of VaMYB4a protein expression in untransformed and VaMIEL1 transgenic grapevine calli. Calli with good protein expression were selected for RT-qPCR analysis. Lane 1: UT; lanes 2-6: VaMIEL1 and VaMYB4a co-expressed calli. CBB staining was used as a loading control. **(C)** RT-qPCR analysis of the relative expression levels of VaMYB4a in UT, VaMIEL1-OE and VaMIEL1-OE/VaMYB4a-OE co-expressed calli. **(D)** RT-qPCR analysis of the relative expression levels of VaMIEL1 in UT, VaMIEL1-OE, and VaMIEL1-OE/VaMYB4a-OE co-expressed calli. Data represent the mean ± SD of three independent experiments. Asterisks indicate statistically significant differences compared to the untransformed control (**p* < 0.05, ***p* < 0.01; one-way ANOVA with post-hoc Tukey test).
